# Radiofrequency Microneedling: Technology, Devices, and Indications in the Modern Plastic Surgery Practice

**DOI:** 10.1093/asjof/ojad100

**Published:** 2023-11-06

**Authors:** Orr Shauly, Troy Marxen, Ambika Menon, Daniel J Gould, Leonard B Miller, Albert Losken

## Abstract

**Background:**

Since the initial invention of microneedling, advancements have been made to improve the desired effects. The addition of radiofrequency to microneedling devices was developed within the past decade as a way to induce thermal injury and increase dermal heating to enhance the dermal wound healing cascade.

**Objectives:**

With an overabundance of literature and mainstream media focused on microneedling and radiofrequency microneedling, this review aims to focus on the available high-quality evidence.

**Methods:**

A comprehensive review of the literature was performed across PubMed (National Institutes of Health, Bethesda, MD) and Embase (Elsevier, Amsterdam, the Netherlands) databases. Attention was focused on manuscripts that provided objective data with respect to clinical application, innovation, anatomy, and physiology.

**Results:**

Optimal outcomes are achieved when needle depth is targeted to the reticular dermis. Needle depth should reflect the relative differences in epidermal and dermal thickness throughout the face. A depth of at least 1.5 mm should be used for the forehead and temporal skin, 1.0 mm for the malar region, 2.0 mm (maximum depth for radiofrequency microneedling) for the nasal side walls, 0.5 mm for the perioral skin, and 1.5 mm for the neck. Deeper settings can be used with care to provide some fat reduction in the submentum.

**Conclusions:**

The authors find herein that radiofrequency microneedling is a safe adjunctive tool to surgical aesthetic procedures. The addition of radiofrequency poses an advance over traditional microneedling devices for skin tightening, with improvements in both safety and efficacy over time.

**Level of Evidence: 5:**

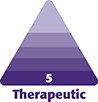

Within the past few decades, microneedling has gained popularity as a technique aimed to improve aesthetic outcomes by decreasing scar burden and diminishing signs of aging, such as rhytids. It was first described as a skin needling procedure to release fibrous strands in the face thought to be responsible for scars and rhytides.^1^ This technique was further investigated by using an empty tattoo gun to dermabrase surgical scars.^2^ More than a decade later, the first true microneedling device was created that functioned by inserting multiple hypodermic needles into the subcuticular layer to break down connective tissue strands and defects.^3^ All devices today stem from this basic principle, with further refinement of needle gauge, distribution, density, angle, and depth, with the recent addition of radiofrequency as an additional means of collagen disruption. Microneedling has tremendous potential; however, it is vital to recognize the efficacy and role of the plethora of available devices on the market today.^4^

Microneedling relies on repetitive skin punctures using needles, which attempts to elevate the underlying defect to improve the contour of the skin.^5^ This is possible by releasing the depressed area from the connective tissue beneath and initiating cutaneous microwounds to establish the connective tissue healing process in a new, smooth plane. The aim is to restore contour to the overlying skin.^1^ There is a balancing act required in performing microneedling that is dependent upon controlled, focused injury. A key component of this technique is sparing the epidermis while simultaneously stimulating dermal inflammation, proliferation, and revascularization. This process, with the help of numerous growth factors, remodels the dermis.^6,7^ Fibroblasts are recruited to the zone of injury and proliferate, which, in combination with angiogenesis, creates a fibronectin matrix for laying down new collagen.^8^ Essentially, a percutaneous collagen induction process, the end result aims to tighten the skin and improve the aesthetic outcome by lining the region with new collagen that was formerly vacant from atrophic scarring.^9^

Since the initial invention of microneedling, advances have been made to improve the desired effects.^10^ The addition of radiofrequency to microneedling was developed within the past decade as a way to induce thermal injury and increase dermal heating to enhance the dermal wound healing cascade.^11^ Such thermal injury can lead to fibrotic remodeling. Fibrosis of the tissue leads to the added tightening effects of radiofrequency microneedling when compared with traditional microneedling. Radiofrequency has also been shown to initiate re-epithelialization, and coagulated columns in the dermis have shown mixed cellular infiltration, angiogenesis, and the formation of granulation tissue as soon as 4 days after treatment.^12^ In contrast to lasers, insulated radiofrequency microneedling delivers a focused, local injury to the dermis, sparing epidermal injury that can cause undesirable erythema, changes in pigmentation, and scarring that is often seen with laser treatments and traditional microneedling.^13^

The ultimate goal of radiofrequency microneedling is to trigger the formation of new skin at the level of the dermis. This is important to bear in mind when evaluating available devices and innovative new technology. Percutaneous collagen induction fills atrophic scars, causing textural skin changes, reducing scar burden, and minimizing rhytids, while avoiding changes in pigmentation that may occur by way of lasers or peels.^11^ Optimal outcomes with radiofrequency microneedling are thus achieved when needle depth is adjusted to reach the dermis, sparing the effects of radiofrequency on the epidermis, with targeted thermal injury to reticular collagen. It is in this layer that scarring causes tightening of the tissues and overlying epidermis. Superficial techniques may lack in achieving the desired effects of skin tightening.

With an overabundance of literature focused on microneedling and radiofrequency microneedling, this review aims to focus on the high-quality evidence behind radiofrequency microneedling. This review will also provide a concise summary of available devices, cost and appropriate administration, and the most effective use of each in clinical practice.

## METHODS

The primary endpoint of this review was to study the pathophysiology of radiofrequency microneedling, and report on all available devices, operating parameters, and reported outcomes and complications. Secondary endpoints of this included reviewing the reported efficacy of available devices and providing an objective summary of the clinical application of these devices for operator use. The inclusion criteria were those articles that included either the primary or secondary endpoints of the study. The exclusion criteria were those articles that were industry-funded, non-English, letters, editorials, or any article that did not discuss with objective data any of the endpoints of this study.

PubMed (National Institutes of Health, Bethesda, MD) and Embase (Elsevier, Amsterdam, the Netherlands) databases were used to identify primary and secondary manuscripts in the context of this literature review, with a focus on radiofrequency microneedling devices, efficacy, complications, and indications for use. Primary articles were defined as those in which the radiofrequency microneedling outcomes were of primary focus, whereas secondary articles contained relevant information about the described procedure or product without clinical data. The references of manuscripts included in this review were studied for additional relevant papers. Select historical texts and textbook chapters were also included to provide well-established and critical evidence for this review. Additional searches were conducted to retrieve relevant papers on the detailed physiology of radiofrequency microneedling and the proposed mechanism of action with the addition of radiofrequency.

### Literature Search

A literature search was performed in PubMed by O.S. with the following search terms: ((Radiofrequency Microneedling) OR (“Radiofrequency Resurfacing”) OR (“Microneedling”[Mesh]) OR (“Patient Outcome Assessment”[Mesh]) OR (Patient Reported Outcomes)) AND (microneedling) AND (radiofrequency). No limits were implemented in the search query.

### Study Selection

All studies published in or after 2010 were evaluated. Author T.M. independently assessed the selected studies in 2 rounds based on specific search criteria—inclusion criteria included reported outcomes, complications, clinical use, or pathophysiology. Exclusion criteria included those studies that did not specify devices when reporting outcomes or did not specify use parameters in addition to those discussed above. If in the first round, inclusion or exclusion criteria could not be assessed from just the title or abstract, the full text was surveyed for the inclusion or exclusion criteria. O.S. and D.J.G. were consulted if the inclusion criteria were not explicitly met, but the study did not meet any of the exclusion criteria. The methodological quality of the studies included in this review was assessed using the Cochrane Collaboration (London, UK) tool for assessing the risk of bias.

### Data Collection

Data from each study were extracted into a form with the following parameters: primary author, publication year, study design, number of patients, mean age, device used, aesthetic target, technique, outcomes, and complications.

## RESULTS

### Microneedling Depth

**Figure 1.**
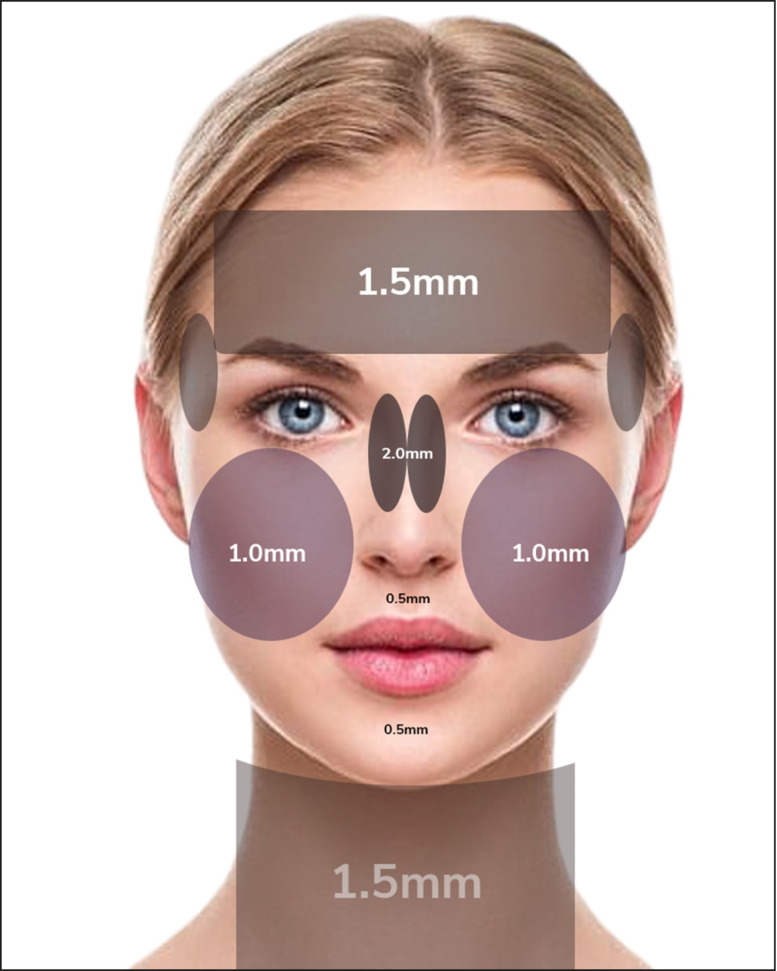
Microneedling depth guidelines for regions of the face.

Optimal outcomes are achieved when needle depth is targeted at the reticular dermis. A topographic study of epidermal and dermal thickness was performed and published epidermal and dermal relative thickness (eRT and dRT) values throughout the face.^14^ Chopra et al found that the epidermis was thinnest at the posterior auricle (1.0 eRT) and thickest at the upper lip (2.12 eRT). This translates to an epidermal thickness (in mm) ranging from ∼0.06 to 0.03 mm. The aforementioned cadaveric study also quantified dermal thickness, with the thinnest dermis at the upper eyelid (1.0 dRT) and the thickest at the nasal side walls (2.59 dRT). This corresponds to a dermal thickness ranging from ∼0.8 to 2.0 mm. This is consistent with studies that demonstrate that needle depth should exceed 1 mm, but ideally range from 1.5 to 2.5 mm to reach the reticular dermis throughout the face.^15,16^ These recommendations have been summarized in Figure 1. Needle length has not been shown to increase depth; therefore, the literature does not recommend a specific needle length when evaluating available devices, only the ability to achieve the necessary adjustable depth of insertion.^16^

There are 2 ways to deliver radiofrequency through the device, either through the use of monopolar or bipolar energy flow. Monopolar technology allows the energy to be deposited more deeply in the deep dermis. Bipolar allows for higher levels of energy to be delivered but cannot penetrate as deeply. The addition of radiofrequency creates heat by colliding charged molecules, and penetration of this energy depends on skin hydration, collagen content, thickness, and heat. Importantly, the depth of penetration tends to increase with the lower frequencies of the device being used. Impedance is an important concept, and the user must understand that different tissues have different impedances, where thick dermis, which is well hydrated, has a higher impedance than thin dermis, which lacks robust blood flow and hydration.

### Providers

Advanced practice providers such as nurse practitioners and physician assistants, in addition to nurse injectors, are a valuable addition to practices that may offer microneedling as an adjunctive aesthetic tool. Studies have demonstrated that microneedling is a safe procedure and can be performed effectively by nurse injectors, nurse practitioners, or physician assistants in this practice setting with appropriate supervision.^17-19^

Aestheticians offer microneedling services for rejuvenation of the skin, but often do not exceed depths of 0.5 mm as most states do not permit depths exceeding that of the epidermis unless further educated or certified. This has not been shown to be an effective depth for adequate scar revision, a reduction in the depth of rhytids, or potent rejuvenation of the skin, as discussed previously. However, under supervision, certified injectors, nurse practitioners, and physician assistants may safely treat at any depth.

Microneedling can be performed in successive treatment sessions until the desired aesthetic outcome is achieved. Studies demonstrate that it may be advisable to have repeat sessions performed by a board-certified physician, as concerns such as increased pain may arise on repeat sessions.^20^ However, another study demonstrated decreased pain on repeat sessions.^21^ In the context of radiofrequency microneedling, although shown to be effective and safe, additional microtrauma to the reticular dermis should be supervised closely, especially with repeat sessions (Table 1).

**Table 1.** Variables that Dictate the Precise Placement of Needles in the Desired Tissue

Operator technique and experience
Depth chosen on selected device
Characteristic and resistance of target tissues
Needle sharpness (needle length has not been shown to dictate depth of placement)
Density of needles on the selected device
Chosen level of frequency

### Procedure Parameters

While microneedling is a safe procedure, there are also parameters that can be followed to help guide the technician or surgeon (Table 2). The presence of pinpoint bleeding can be used as an endpoint for the procedure.^22^ Thus, it is expected, and not uncommon, for transient erythema to occur between 1 and 7 days after the procedure.^23-26^ The most commonly reported complications of microneedling consist of mild pain, swelling, and dyspigmentation.^26-30^ Dyspigmentation is a rare side effect that is mainly a concern for darker pigmented patients who may experience postinflammatory hyperpigmentation as the thermal energy can be placed too close to the epidermal layer or may be due to postprocedural ultraviolet exposure. Special precaution should be advised to avoid ultraviolet exposure in the following days to weeks after undergoing microneedling.^4^

**Table 2.** Clinical Pearls: Periprocedure Recommendations

Preprocedure: informed consent and photographs with and without flash of both sides of the face at 0°, 45°, and 90°, both with and without smiling (total 20 photographs)
Periprocedure: treated area cleansed and topical anesthetic of choice used (commonly BLT/LT gel).^a^ Topical anesthetic is removed prior to treatment
Periprocedure: maintain sterility of the skin with alcohol or chlorhexidine wipes
Postprocedure: topical clindamycin, hypochlorous sodium, bacitracin

^a^Betacaine–lidocaine–tetracaine, or lidocaine–tetracaine gel or cream.

Different modalities can be used in the administration of microneedling. These include a pen, roller, stamp, and radiofrequency. There are variable rates of complications between modalities; however, all of the modalities have been shown to be safe.^22^ It should be noted that special caution should be given to patients on blood thinners and those with coagulation disorders, as these patients have often been excluded from study cohorts, and further examination is required before safely using microneedling in this group.

Energy parameters were demonstrated to be important in dictating outcomes. In several studies, higher energy treatments and devices yielded better on-table and long-term clinical results. Energy parameters should be tailored to the area of concern. For radiofrequency microneedling of the periorbital area, radiofrequency (RF) energy levels should be between 15 and 30.^31^ For the neck, RF energy levels should remain between 20 and 40.^31^ For the body, levels should be between 25 and 40,^31^ to prevent increased side effects such as skin redness, hash marks, pain, and therefore, longer interval between treatments. There are few studies that investigate pulse duration—the literature recommends that a duration be set to a minimum of 300 to 500 ms but ranging as high as 2800 to 4000 ms in most cases, as this allows enough time to transmit sufficient heat through the tissues.

Postoperatively, patients are instructed to avoid all make-up products for 24 to 48 h and apply a facial cleanser before bed and in the morning. In addition, across some reported protocols, patients were recommended to apply topicals commonly used after ablative laser treatments (practice-dependent preference). It is also best to avoid sun exposure for 2 weeks, as reported across all studies. If the face is treated, it is recommended to elevate the head of bed for 48 to 72 h to decrease postprocedure edema.

### Short-Term Complications

There are several short-term complications that can result from fractional radiofrequency microneedling, none of which are serious or life-threatening (Table 3). Pain is common as a result of the needling mechanism, but it is relatively mild and short lived.^32^ Pain during the procedure can be mitigated with adjuncts, including nerve blocks, superficial skin blocks, tumescent anesthesia, or oral narcotics or anxiolytics, to name a few.^13^ There may also be erythema and edema in the surrounding area for several hours, up to days following the procedure.^22^ While pinpoint bleeding is often used as a desired endpoint, if surpassed, bruising can also occur, especially if the procedure is performed in a sensitive area or around the eyes.^22,33-35^ Rates of superficial infection following microneedling remain low, especially if the needles are kept sterile; however, as with any procedure that involves penetration through the epidermis, there is still a chance of infection.

**Table 3.** Common Postprocedure Complications

Complication	Prophylaxis
Superficial infection	Sterile procedure, postoperative oral antibiotics, or topical clindamycin ointment
Acne flair, perioral herpes, periocular herpes, if area is treated (eyes, mouth) and the patient has a history of HSV-1 outbreaks in treatment region	Preprocedure and postprocedure prophylactic acyclovir
Persistent grid marks	Lower energy in subsequent treatments
Redness and edema	Postprocedure elevation, topical cleansers. Topical or oral antihistamines
Postinflammatory hyperpigmentation	Lower energy and pulse duration. Topical bleaching agents like hydroquinone, azelaic acid, TXA in combination with tretinoin, or kojic
Postprocedure bumps (from needle entry points)	Topical steroid (triamcinolone) with combination tretinoin

### Efficacy

The efficacy of radiofrequency microneedling has been investigated in a plethora of studies, with aesthetic targets varying from periorbital wrinkles, other facial rhytids, acne, and photoaging (Table 4).^32-42^ Radiofrequency microneedling is currently efficacious in the treatment of aging facial rhytids, acne scars, cellulite, striae, rosacea, alopecia, and axillary hyperhidrosis.^9,43-45^ There are myriad available products (Table 5).^43-58^

**Table 4.** Efficacy of Radiofrequency Microneedling

Study	Technique	Aesthetic target	Summary/outcomes
Kim et al^33^	Fractional radiofrequency microneedling	Periorbital wrinkles	Significant reduction in wrinkles and improvement in satisfaction
Lee et al^34^	Fractional radiofrequency microneedling	Periorbital wrinkles	All patients were satisfied with treatment and most received clinical and photographic assessment improvement
Kwon et al^35^	Monopolar radiofrequency microneedling	Periorbital wrinkles	Long microneedling is preferable to short microneedling and results in significantly improved photographic assessments
Jeon et al^36^	Fractional radiofrequency microneedling	Periorbital wrinkles	Long-term results for improvement of periorbital wrinkles are superior in microneedling in comparison to botulinum toxin
Gold et al^37^	Fractional radiofrequency microneedling	Mid- and lower face laxity and wrinkles	Significant wrinkle reduction, skin tightening, and lifting of the mid- and lower face resulted from the treatment
Alexiades et al^38^	Bipolar fractional radiofrequency microneedling	Face and neck rhytides and laxity	Temperature-controlled bipolar radiofrequency results in high levels of patient satisfaction, and significant reduction in wrinkles and skin laxity
Zhang et al^39^	Fractional radiofrequency microneedling	Facial photoaging	There were high levels of patient satisfaction and significant improvements in 2 measures of facial photoaging (rhytides, skin tightening, complexion)
Liu et al^40^	Fractional radiofrequency microneedling	Rhytids and facial laxity	3-Dimensional imaging revealed improvement in roughness parameters as an indication of treatment efficacy in photoaging
Seo et al^41^	Fractional radiofrequency microneedling	Skin rejuvenation	Objective measures (skin hydration, roughness, histological findings dermal thickness, collagen content) and subjective measures (patient satisfaction) all improved after treatment
Alexiades et al^42^	Fractional radiofrequency microneedling	Facial and neck rhytides	Treatment had a 100% response rate, identifying an optimal dermal temperature of 67°C with a duration of 3 to 4 s
Lu et al^43^	Fractional radiofrequency microneedling	Nasolabial and infraorbital rhytides	Treatments targeting the deep dermal layer were more effective than superficial, and nasolabial folds responded to treatment more than infraorbital rhytids

**Table 5.** Available Products

Device	Summary
Endymed (Caesarea, Israel)^44^	Multigenerator radiofrequency device that implements an array of radiofrequency sources and controls the phase of the current flowing through the electrodes using an algorithm
Infini (Infini Sonic Therapy, Van Nuys, CA)^45^	Fractional radiofrequency microneedling device that includes insulated, preset needle depths and independently variable power levels and uses a combination of radiofrequency energy and microneedling
Morpheus8 (InMode, Irvine, CA)^46^	Fractional radiofrequency microneedling device that includes dual treatment modes (cycle and fixed) as well as insulated microneedle tips delivering energy at variable depths for different target regions
SkinPen (Crown Laboratories, Inc., Johnson City, TN)^47^	Microneedling device that includes 14 solid 0.25 mm needles operating at a speed between 6300 and 7700 rpm with maximum needle cartilage extension <2.5 mm
Profound (Candela Medical, Wayland, MA)^44^	Bipolar radiofrequency device consisting of 2 electrodes emitting a fast, alternating current at a predetermined distance by means of 32-gauge microneedles into the reticular dermis
Dermaroller (Dermaroller GmbH, Wolfenbüttel, Germany)^48^	A simple drum shaped derma roller with 8 rows bearing 192 fine needles with a length of 0.5 to 2.0 mm and a diameter of 1.0 mm designed for easy use and delivery/penetration through the stratum corneum *This is not a radiofrequency device but is often used in addition to the other devices mentioned in this table.
Secret (Cutera, Inc., Brisbane, CA)^49^	An innovative fractional radiofrequency microneedling system with different size treatment tips (25, 64 pins) designed to stimulate and remodel collagen from the inside out. Delivers precise, controlled energy at various depths
Fractora (InMode)^50^	A novel 24-pin coated tip fractional radiofrequency microneedling device delivering various levels of radiofrequency energy at predetermined depths
Vivace (Aesthetics Biomedical, Inc., Phoenix, AZ)^51^	A fractional radiofrequency microneedling device that uses bipolar radiofrequency energy using a noninsulated cartridge with 36 needles of 0.3 mm diameter, in combination with light-emitting diode light
Intracel (Perigee Medical LLC, Park City, UT)^52^	A radiofrequency microneedling device with a 1 cm^2^ tip that contains 49 microneedle electrodes that can vary in depth and energy delivered to the tissue
Genius (Lutronic, Billerica, MA)^53^	A fractional radiofrequency microneedling device that improves on previous generations of devices by including adjustable power, depth, exposure time to reduce zones of injury, and deliver highly targeted energy at depths of 0.5 to 3.4 mm
Voluderm (Lumenis, Yokneam Illit, Israel)^54^	A fractional radiofrequency microneedling device emphasizing nonmechanical insertion using 1.00 mm long × 0.15 mm diameter ultrathin electrode tips to mitigate perceptible skin penetration
Potenza (Cynosure, Westford, MA)^55^	A versatile fractional radiofrequency microneedling device that can utilize monopolar or bipolar settings, as well as single or multineedle configurations for rhytides, acne, skin tightening, or sun damage

### Topical Treatments

Microneedling has been used in conjunction with skin treatments such as trichloroacetic acid. Multiple studies have reported evidence for improvement of atrophic acne scarring after using this combination treatment method.^24,59,60^ Another study found evidence for significant aesthetic improvement in the treatment of infraorbital dark circles with this combination treatment.^61^

Other topical treatments are utilized in conjunction with microneedling, including exosome therapies, including adipose tissue stem-cell-derived exosomes, topical medications are not only delivered after microneedling in scar treatment but also in facial rejuvenation.^62^ In the future, many cell-based therapies and additional medications will be utilized at the time of microneedling to improve skin quality. However, FDA approval is still pending for any clinical use in this setting, with no FDA-approved exosome products on the market.

Although there is limited data looking at the use of topical treatments with radiofrequency microneedling, the efficacy and safety profile of the utilization of topicals with microneedling indicate that there may be promising results in the use of radiofrequency microneedling in conjunction with topical skin treatments.

## DISCUSSION

The Profound (Candela Medical, Wayland, MA) bipolar radiofrequency microneedling system is able to penetrate and deliver thermal energy by means of 32-gauge microneedles into the reticular dermis, as reported in the literature. This process initiates the fibroblast cascade, increasing collagen, elastin, and hyaluronic acid, resulting in improved aesthetic outcomes.^58^ Morpheus8 (InMode, Irvine, CA) is a dual treatment mode delivery with multipolar radiofrequency delivery. It allows for controlled depth delivery at 4, 3, 2, and 1 mm on the facial device and up to 8 mm depth on the body device with targeted settings for multilevel deployment and pulse delivery at each depth. This device has been well studied in the dermatologic literature (Video 1, available online at www.aestheticsurgeryjournal.com).

**Figure 2.**
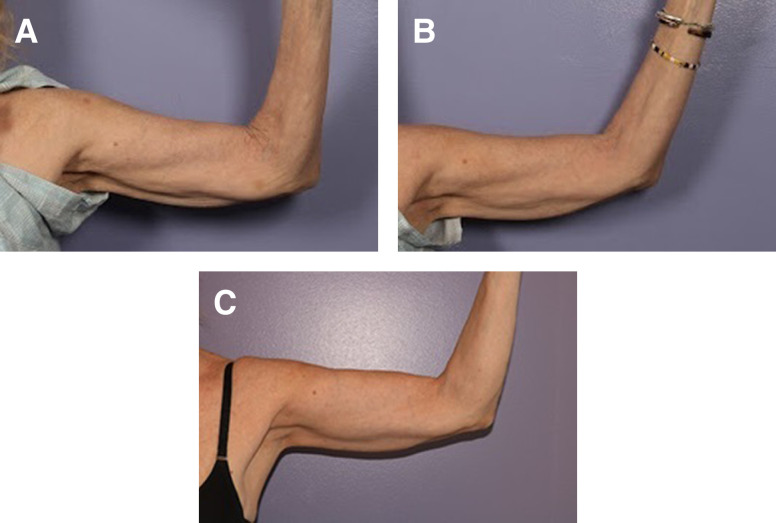
This is a 60-year-old female with concomitant facelift, desires radiofrequency microneedling to the arms at the same time at (A) prior to treatment, (B) 2 weeks posttreatment, and (C) 6 months posttreatment with 2800 ms pulses at 4 mm—40 depth and energy setting.

Some general settings include the use of different energy settings at different depths, a general rule of delivery is 4 mm—40, 3 mm—30, 2 mm—20, and 1 mm—10 for the energy settings. Note that as the depth decreases, the energy needed decreases because the impedance is higher in the dermis, compared to the deeper subdermal tissue which contains fat. The operator should always be thinking about the depth of the needle and the energy needed to induce the thermal injury sufficient for change and activation of the healing cascade. For example, when performing radiofrequency microneedling on the arm, consideration for tougher skin with higher impedance requires high energy settings (Figure 2).

Microneedling can be safely used in conjunction with skin treatments such as trichloroacetic acid and other mild-to-moderate chemical peels. The authors recommend limiting concentration to <30% so as to not penetrate too deeply in conjunction with the radiofrequency microneedling inflammatory reaction. This has been reported to be successful in postacne scarring and has shown significant aesthetic improvement in the treatment of infraorbital dark circles in this combined approach. Combination peel and radiofrequency microneedling may also be used to target hyperpigmented scars.

Overtreatment is possible, in that an injury that overcomes the healing response can lead to poorer outcomes, so there is a sweet spot for treatment algorithms, but based on average-sized faces, the energy and pulses required to treat the square centimeters at the correct depths translates to somewhere between 1200 and 2000 pulses total in most faces and 2000 and 3000 to the trunk, back, arms, and legs.

Most common complications, including those arising from overtreatment, can easily be addressed in the clinic. Superficial infection can be treated with postoperative oral antibiotics or a topical clindamycin ointment. In patients who return for repeat radiofrequency microneedling sessions, prophylactic clindamycin ointment may be used postoperatively. Persistent grid marks are a common complication that can simply be addressed by using a lower energy setting in subsequent treatments. The “persistence” of grid marks usually only lasts for 1 to 2 weeks following the higher energy treatment. Postprocedure bumps from needle entry are common in the first 24 to 48 h and can be addressed by topical low-dose steroid with a combination of tretinoin in patients with the most inflammatory reactions. In the authors’ practice, postprocedure topical clindamycin is used in all patients, and hypochlorous sodium solution postmicroneedling helps neutralize the inflammatory reaction and decrease the need for postprocedure topical steroids (Video 2, available online at www.aestheticsurgeryjournal.com).

When using dual radiofrequency and microneedling devices, given the data presented herein, the authors recommend adjusting needle depth to reflect the relative differences in epidermal and dermal thickness throughout the face. A depth of at least 1.5 mm should be used for the forehead and temporal skin, 1.0 mm for the malar region, 2.0 mm (maximum depth for radiofrequency microneedling) for the nasal side walls, 0.5 mm for the perioral skin, and 1.5 mm for the neck.

It is important for plastic surgeons to understand this technology and what treatment modalities are efficacious for our patients. It is also important to note that radiofrequency microneedling is safe when combined with other procedures including rhytidectomy, blepharoplasty, brow lifts, and other surgical interventions. Skin resurfacing technologies with either ablative or nonablative lasers target the epidermis, whereas radiofrequency microneedling devices (when performed correctly) do not disrupt this layer. As such, radiofrequency microneedling is more effective at targeting the underlying pathogenesis of many skin disorders, as summarized herein.

One of the authors recommends the combination use of the Profound device and SkinPen (Crown Laboratories, Inc., Johnson City, TN) for facial cosmesis, as this has been noted to be most effective for targeting most aspects of the face excluding the perioral skin. The dermaroller is recommended for the use on perioral skin, as needles can preferentially target the 0.5 mm depth. Another author recommends the Morpheus8 device for patients with demonstrated sensitive epidermis, as the insulated needles and radiofrequency only function spare this layer or any trauma altogether. This is excellent for patients with acne scarring or a history of hypertrophic and keloid scarring. This is also preferentially used for nonfacial radiofrequency microneedling, as it provides the most flexibility with needle depth and length and energy delivery.

Due to the safety profile of these devices, when used in the right hands, they are a well-tolerated treatment modality with few complications, discussed above. As summarized herein, it was found that the most commonly reported side effects were pain, erythema, swelling at times, and persistent grid marks that unequivocally resolved after several weeks. Erythema was transient and often resolved with photoprotection and skin care. The highest conferred risk of complications was user error, thus requiring adequate training and supervision. The authors therefore advocate for surgeons and physicians to take ownership of this valuable treatment modality.

## CONCLUSIONS

The authors find herein that radiofrequency microneedling is safe and well tolerated in the treatment of a plethora of skin disorders, when in the right hands. It is important for plastic surgeons to remain at the forefront to maintain proper patient safety and treatment efficacy.

## Supplemental Material

This article contains supplemental material located online at www.asjopenforum.com.

## Supplementary Material

ojad100_Supplementary_Data
